# Intraoperative transit-time flowmetry in patients undergoing coronary surgery to determine relationships between graft flow and patency and prior coronary interventions and flow demand: a retrospective study

**DOI:** 10.1186/s13019-018-0806-6

**Published:** 2018-11-22

**Authors:** Hiroyuki Nakajima, Akitoshi Takazawa, Akihiro Yoshitake, Masato Tochii, Chiho Tokunaga, Jun Hayashi, Hiroaki Izumida, Daisuke Kaneyuki, Toshihisa Asakura, Atsushi Iguchi

**Affiliations:** Department of Cardiovascular Surgery, Saitama Medical University, International Medical Center, 1397-1 Yamane Hidaka, Saitama, 350-1298 Japan

**Keywords:** Off-pump, Coronary artery bypass graft, Transit-time flowmetry, Graft flow, Flow demand, Percutaneous coronary intervention

## Abstract

**Background:**

The aim of this study was to delineate impacts of percutaneous coronary intervention (PCI), flow demand, and status of myocardium on graft flow.

**Methods:**

We retrospectively assessed 736 individual coronary artery bypass grafts that had been created as the sole bypass graft for a vascular region in 405 patients. The grafts comprised 334 internal thoracic artery (ITA) to left anterior descending (LAD), 129 ITA and 65 saphenous vein grafts (SVG) to left circumflex (LCX), and 142 gastroepiploic artery (GEA) and 66 SVG to right coronary artery (RCA). Minimal luminal diameter, size of revascularized area, history of myocardial infarction, and PCI in the relevant area were examined to determine whether these factors are associated with flow insufficiency (FI), which was defined as ≤ 20 mL/min.

**Results:**

FI developed in 123/736 grafts (16.7%) and correlated significantly with stenosis in the distal portion (23.0% vs. 12.8%, *p* = 0.0003). Prior myocardial infarction significantly correlated with FI in GEA–RCA (*p* = 0.002) and ITA–LCX grafts (*p* = 0.04). There was a history of PCI to the LAD (PCI group) in 54 ITA to LAD bypass grafts (16.2%), whereas the remaining 280 had no history of PCI to the LAD (no-PCI group). Graft flow was significantly greater in the no-PCI than in the PCI group (53 ± 29 vs. 42 ± 27; *p* = 0.006). The incidences of FI and graft failure were significantly higher in the PCI than the no-PCI group (22.2%, vs. 8.2%; *p* = 0.003; 9.2% vs. 1.8%; *p* = 0.003, respectively).

**Conclusions:**

Prior PCI has a negative impact on graft flow. The influences of small revascularized area, myocardial infarction, and PCI are greater, necessitating consideration of factors associated with flow demand or microvasculature when planning revascularization.

## Background

After coronary artery bypass grafting (CABG), flow to the relevant myocardial area comprises the sum of graft flow and native coronary flow. Prediction of native coronary flow has been improved by evaluating the severity of stenosis in the native coronary artery, for example, by measuring fractional flow reserve (FFR). FFR, which is calculated by measurement of intraluminal pressure, represents the ratio of maximal blood flow through the stenosis to theoretically normal maximal flow, and reportedly reliably detects myocardial ischemia. If myocardial flow demand in the relevant area is smaller than a certain amount, graft flow may be insufficient to achieve long-term patency, irrespective of FFR value. The impact of flow demand has not yet been fully delineated.

Graft flow is commonly measured by transit-time flowmetry (TTFM) intraoperatively. Several recent retrospective observational studies have found that TTFM correlates significantly with graft patency in the early [1] and mid-term [2–4]. We have previously reported that the risk of graft failure increases fourfold or more when graft flow as measured by TTFM is insufficient [5].

In the present study, we examined the characteristics of target coronary lesions and status of revascularized areas to determine the mechanisms underlying graft flow insufficiency and the impacts of flow demand and peripheral vasculature on graft flow and patency.

## Methods

We reviewed the clinical records and angiograms of 405 patients with 1284 bypass grafts who had undergone off-pump CABG and had postoperative coronary angiograms between 2007 and May 2015. They comprised 315 men and 90 women with a mean age of 67 ± 9 years. Postoperative coronary angiography had been performed in all 405 patients (Table 1).

**Table 1:** Baseline patients’ characteristics

No. of patients	405
Age (yrs)	67 ± 9
Male/Female	315 / 90
Hypertension	244 (64%)
Hyperlipidemia	218 (58%)
Diabetes	201 (50%)
Atrial Fibrillation	21 (7%)
Intraaortic balloon pump	55 (17%)
Ejection fraction of LV (%)	55 ± 16
Ejection fraction of LV < 40%	65 (15%)
Total distal anastomoses	1284
Targets per patient	3.2 ± 1.0

LV; left ventricle

To minimize bias, we selected the 736 of these patients’ bypass grafts that were individual and created as the sole bypass graft for the relevant vascular region. These patients were consecutive after exclusion of those without eligible bypass grafts. The selected grafts comprised 334 in situ internal thoracic artery (ITA) to left anterior descending (LAD), 129 in situ ITA and 65 aorto-coronary saphenous vein grafts (SVG) to the left circumflex (LCX), and 142 in situ gastroepiploic artery (GEA) and 66 aorto-coronary SVG to the right coronary artery (RCA). This retrospective study was approved by our Institutional Review Board, which waived the requirement for written informed consent because this was a retrospective observational study.

Our standard procedure has been off-pump CABG using the ITA and GEA. At the beginning of the study period, we preferred to use arterial grafts irrespective of the severity of stenosis. However, we have increasingly used aorto-coronary vein grafts for the LCX and RCA when the stenosis seems to be moderate. We performed preoperative quantitative coronary angiography for all targets of the 736 bypass grafts, measuring the minimal luminal diameter (MLD; measured at the narrowest stenotic lesion proximal to the anastomotic site) and its reference diameter. We categorized the location of stenosis as proximal or distal to indicate the size of myocardial flow demand in the revascularized area. We defined proximal lesions as stenosis at #1–3, 5, 6 and 11, and distal lesions as stenosis at #4, 7 and 12–14. We defined a history of percutaneous coronary intervention (PCI) as any catheter procedure for treating coronary artery disease, even it had been unsuccessful. We defined myocardial infarction (MI) as diagnosis by a cardiologist or the presence of Q-wave on an electrocardiogram and asynergy on echocardiography in the relevant area. PCI and MI were recorded for each vascular region.

After completion of anastomosis, we measured graft flow by using a transit time flow meter (Medi-Stim AS, Oslo, Norway) at approximately 100 to 120 mmHg of systolic arterial pressure with minimal or no inotropic support. We usually administered papaverine or another vasodilator. We defined flow insufficiency (FI) as 20 mL/min or less as measured by intraoperative TTFM and graft failure as occlusion or string sign on postoperative angiography. When we identified significant difference in the incidence of FI between higher and lower values, we defined the value with lowest p as the cuff-off MLD. As we have previously reported, FI correlates significantly with future failure of ITA, GEA, and SV grafts [5].

### Statistical analysis

We have expressed continuous variables as mean ± standard deviation and compared them by unpaired Student’s *t*-test. We compared data of two independent groups by the χ^2^ test. The mean duration of follow-up was 10 ± 14 months. We considered differences in outcomes statistically significant when the *p* value was less than 0.05.

## Results

FI developed in 123/736 (16.7%) grafts and there were 47/736 (6.4%) graft failures. The incidence of FI according to characteristics of the target vessel, bypass graft, revascularized area, and prior MI and PCI is shown in Table 2. For ITA to LAD bypass grafts, the incidence of FI in ITA to LAD with prior PCI was 22.2% (12/54), which is significantly higher than the 8.2% (23/280) in patients without prior PCI (*p* = 0.002). The incidence of FI for distal lesions was 15.0% (20/133), which is significantly higher than that for proximal lesions, namely 7.5% (15/201) (*p* = 0.03). Presence of prior MI did not correlate with FI in LAD.

**Table 2:** Flow insufficiency according to characteristics of target vessel, bypass graft and stenosis location

Target vessel	Bypass graft	(n)	Flow insufficiency	MI (+)	MI (−)	PCI (+)	PCI (−)	Distal lesion	Proximal lesion
LAD	in-situ ITA	334	10.5%(35/334) *	7.0%(3/43)	11.0%(32/291)	22.2%(12/54)	8.2%(23/280)	15.0%(20/133)	7.5%(15/201)
				*p* = 0.42		*p* = 0.002		*p* = 0.03	
LCX		194	18.0%(35/194) **	28.6%(4/14)	17.2%(31/180)	15.0%(3/20)	18.4%(32/174)	27.7%(26/94)	9.0%(9/100)
				*p* = 0.29		*p* = 0.71		*p* = 0.0007	
	in-situ ITA	129	24.0%(31/129)	36.4%(4/11)	22.9%(27/118)	18.8%(3/16)	24.8%(28/113)	37.1%(23/62)	11.9%(8/67)
				*p* = 0.04		*p* = 0.60		*p* = 0.0008	
	aorto-coronary SVG	65	6.2%(4/65)	0%(0/3)	6.5%(4/62)	0%(0/4)	6.6%(4/61)	9.4%(3/32)	3.0%(1/33)
				*p* = 0.65		*p* = 0.60		*p* = 0.29	
RCA		208	25.5%(53/208) ***	45.1%(23/51)	19.1%(30/157)	26.3%(5/19)	25.4%(48/189)	48.7%(19/39)	20.1%(34/169)
				*p* = 0.0002		*p* = 0.93		*p* = 0.0002	
	in-situ GEA	142	26.8%(38/142)	45.9%(17/37)	20.0%(21/105)	30.0%(3/10)	26.5%(35/132)	50.0%(11/22)	22.5%(27/120)
				*p* = 0.002		*p* = 0.81		*p* = 0.007	
	aorto-coronary SVG	66	22.7%(15/66)	42.9%(6/14)	17.3%(9/52)	22.2%(2/9)	22.8%(13/57)	47.1%(8/17)	14.3%(7/49)
				*p* = 0.04		*p* = 0.97		*p* = 0.006	
Overall		736	16.7%(123/736)	28.0%(30/107)	14.8%(93/629)	21.5%(20/93)	16.0%(103/643)	23.0%(65/283)	12.8%(58/453)
				*p* = 0.0007		*p* = 0.19		*p* = 0.0003	

GEA; gastroepiploic artery ITA; internal thoracic artery LAD; left anterior descending artery LCX; left circumflex artery

MI; myocardial infarction PCI; percutaneous coronary intervention RCA; right coronary artery SVG; saphenous vein graft;  * vs. **; *p* = 0.01;  * vs. ***; *p* < .0001

For ITA to LCX and GEA to RCA grafts, the incidence of FI was significantly higher in patients with prior MI in the revascularized area (*p* = 0.04 and p = 0.002, respectively). The incidence of FI was significantly higher when the stenosis was located in the distal portion than that when it was in the proximal portion (*p* = 0.0003).

**Fig. 1:**
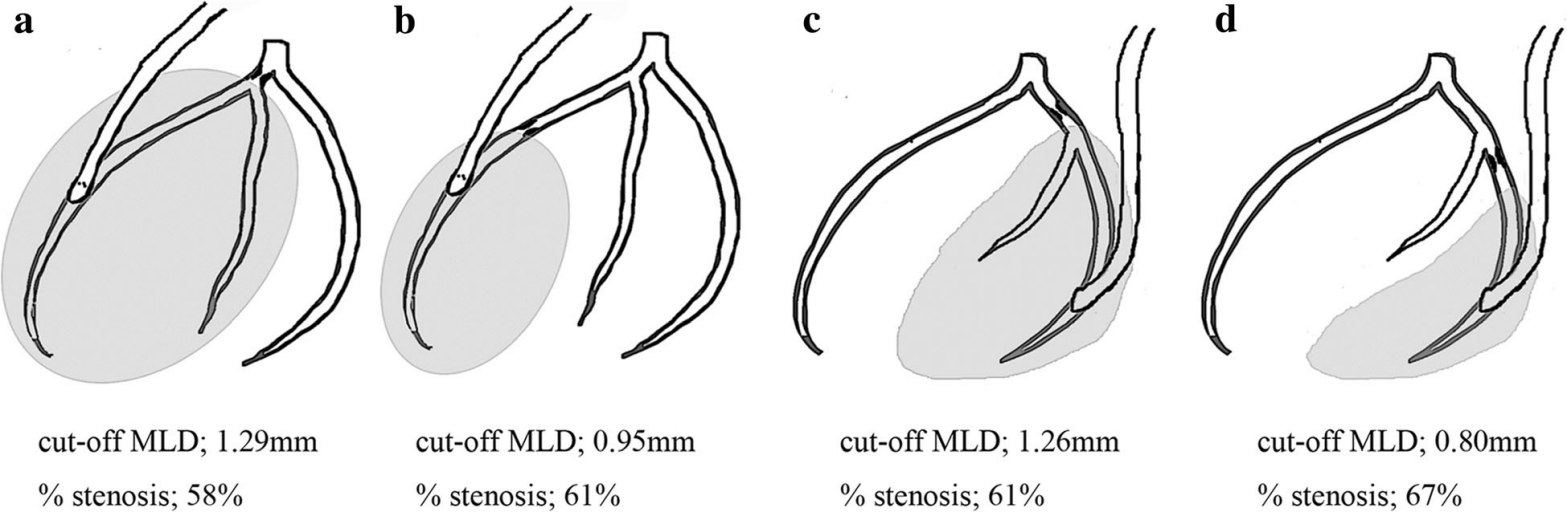
Cut-off values for minimal luminal diameter and calculated % stenosis. The cut-off values for minimal luminal diameter and calculated % stenosis were (**a**) 1.29 mm and 58% for proximal LAD stenosis; (**b**) 0.95 mm and 61% for distal LAD stenosis; (**c**) 1.26 mm and 58% for proximal LCX stenosis; and (**d**) 0.80 mm and 67% for distal LCX stenosis. The smaller the revascularized area, the more severe stenosis is necessary to avoid flow insufficiency. LAD; left anterior descending artery, LCX; left circumflex artery

Reference diameters and cut-off MLDs are shown in Table 3. For ITA to LAD grafts, the cut-off MLD was 1.29 for proximal and 0.95 for distal lesions; for ITA to LCX, 1.26 for proximal and 0.80 for distal lesions; for GEA to RCA, 1.27 for proximal lesions. No cut-off value was identified for GEA to RCA distal lesions. The incidence of FI was 50.0% for GEA-RCA distal lesions, irrespective of severity of stenosis. We were unable to identify a significant cut-off value for SVG to LCX or to RCA grafts. Compared with proximal lesions, cut-off values for MLD and % stenosis were lower by 0.34 mm and 3%, respectively, for the LAD, and by 0.46 mm and 6%, respectively, for the LCX (Fig. 1).

**Table 3:** Flow insufficiency according to MLD higher and lower than cut-off value and stenosis location

Bypass graft	Stenosis location	Reference diameter (mm)	MLD < cutt-off value	Cut-off value (mm)	calculated % stenosis	MLD > cut-off value
in-situ ITA	Proximal	3.07 ± 0.75	8/159	1.29	58%	7/42
			(5.0%)	*p = 0.01		(16.7%)
	Distal	2.42 ± 0.53	10/100	0.95	61%	10/33
			(10.0%)	*p = 0.005		(30.3%)
in-situ ITA	Proximal	3.21 ± 0.84	1/46	1.26	61%	7/21
			(2.2%)	*p = 0.0003		(33.3%)
	Distal	2.40 ± 0.53	7/35	0.80	67%	16/27
			(20.0%)	*p = 0.0005		(59.3%)
in-situ GEA	Proximal	3.00 ± 0.73	22/110	1.27	58%	5/10
			(20.0%)	*p = 0.03		(50.0%)
	Distal	2.79 ± 0.77		N/A		

GEA; gastroepiploic artery ITA; internal thoracic artery LAD; left anterior descending artery LCX; left circumflex artery

MLD; minimalluminal diameter RCA; right coronary artery *; comparison of higher versus lower than MLD

As shown in Table 4, for 54/334 ITA to LAD bypass grafts (16.2%) there was a history of PCI to the LAD (PCI group), whereas for the remaining 280 ITA to LAD grafts there was no history of PCI to the LAD (no-PCI group). Stents had been implanted in 28 of the 54 in the PCI group (51.8%) and PCI had been unsuccessful in 15/54 (27.8%). Graft flow was significantly greater in the no-PCI than in the PCI group (53 ± 29 vs. 42 ± 27; *p* = 0.006). The incidences of FI and graft failure were significantly higher in the PCI than the no-PCI group (22.2%, vs. 8.2%; *p* = 0.003; 9.2% vs. 1.8%; p = 0.003, respectively). Of 54 ITA–LAD bypass grafts in the PCI group, 25 were for distal and 29 for proximal lesions. In the PCI group, graft flow and incidence of FI and graft failure were 35 ± 24, 8/25 (32.0%), and 3/25 (12.0%), respectively, for distal lesions, whereas they were 49 ± 29 (*p* = 0.03), 4/29 (13.8%) (*p* = 0.10), and 2/29(6.9%) (*p* = 0.51), respectively, for proximal lesions.

**Table 4:** Graft flow and angiographic results according to prior history of percutaneous coronary intervention

		PCI (+)	PCI (−)	*p* value
Number of patients		54	280	–
Age		67 ± 9	67 ± 10	0.44
Female		12 (22.2%)	68 (24.3%)	0.75
DM		25 (46.3%)	149 (53.2%)	0.35
Stenosis location and severity	Stenosis at distal portion #7 or #8	25 (46.3%)	107 (38.2%)	0.27
	Minimal luminal diameter (mm)	0.82 ± 0.53	0.73 ± 0.52	0.12
	Reference diameter (mm)	2.76 ± 0.57	2.82 ± 0.78	0.29
	Calculated severity of stenosis (%)	70 ± 19	74 ± 17	0.07
Prior coronary intervention	Stent implantation	28 (51.8%)	–	–
	Drug-eluting	18	–	–
	Bare metal / unknown stent	6 / 4	–	–
	Balloon angioplasty	3 (5.6%)	–	–
	Unsuccessful	15 (27.8%)	–	–
	No detalied information about old PCI	5 (9.2%)	–	–
	PCI complications, such as dissection, perforation, etc.	3 (5.6%)	–	–
Graft flow and angiographic results	Graft flow (ml/min)	42 ± 27	53 ± 29	0.006
	Fow insufficiency (< 20 ml/min)	12 (22.2%)	23 (8.2%)	0.002
	Angiographic competitive flow	5 (9.2%)	3 (1.1%)	< 0.001
	Graft failure	5 (9.2%)	5 (1.8%)	0.003

PCI; percutaneous coronary intervention

## Discussion

Flow demand and peripheral vascular resistance in revascularized areas are the fundamental factors that influence graft flow and patency; however, they have not yet been fully explored, probably because vascular resistance varies as a result of continuously being adjusted according to oxygen consumption and left ventricular work. Thus, vascular resistance cannot be reliably quantified in clinical studies. In an attempt to circumvent these difficulties, we examined location of stenosis (distal vs. proximal) and history of MI and PCI in the relevant area, all of which are presumably associated with the status and size of revascularized myocardial areas and flow demand, to determine how these factors influence graft flow and patency.

Competitive flow can be avoided by appropriate target assessment, graft selection, and configuration [6, 7]. Functional assessment of native coronary stenosis, such as by assessing FFR or coronary flow velocity reserve (CFVR), has raised some issues. Van de Hoef and colleagues reported that results of FFR and CFVR were discordant in 31% or 37% of target vessels at cut-off FFR values of 0.75 or 0.80, respectively [8]. This discordance is characteristic of microvascular disease (MVD); adverse cardiac events and deaths were significantly associated with normal FFR and abnormal CFVR in that study [8]. Additionally, in patients with multivessel disease, including chronic total occlusion (CTO), FFR in collateral donating branches can be overestimated [9].

In the present study, we assessed native coronary stenosis by using quantitative coronary angiography to ascertain the MLD and reference diameter, both of which are traditional but standard measures. Additionally, off-pump CABG favours use of TTFM [10] because cardiopulmonary bypass can reduce systemic vascular resistance and increase graft flow by inducing a hyperaemic state, thus creating a major bias in flow measurements [11]. Moreover, to minimize any bias caused by bypass grafts or targets, we excluded all sequential and composite graft and bypass grafts that were not the sole bypass grafts in the relevant vascular region. For example, when there was a bypass graft to a diagonal branch, we excluded ITA to LAD bypasses in case of any negative interactions [12].

We found that FI was significantly associated with distal lesions, history of MI in the LCX and RCA areas, and history of PCI in the LAD. The cut-off values were higher by 3% for LAD and 6% for LCX for distal lesions than for proximal lesions. These findings suggest that severity of stenosis and CABG strategy should be modified according to whether the stenosis is located distally or proximally and the size of the area to be revascularized. Competitive flow that is attributable simply to moderately stenotic native targets does not remain the primary mechanism for FI at later stages.

We were unable to determine cut-off values for SVG to LCX and RCA grafts, in the case of SVG to LCX possibly because SVG patency is not influenced by the severity of stenosis and there were too few grafts with FI to demonstrate a significant difference. In comparison, as shown in Table 2, for distal RCA, the incidence of FI in SVGs was as high as 47.1%, which is comparable to that for GEA, irrespective of MLD. SVGs have been widely accepted as providing high pressure capacity and being more reliable than arterial grafts for targets with moderate stenosis. The results of this study suggest that SVG is reliable irrespective of stenosis severity provided that flow demand in the grafted area is adequate.

**Fig. 2:**
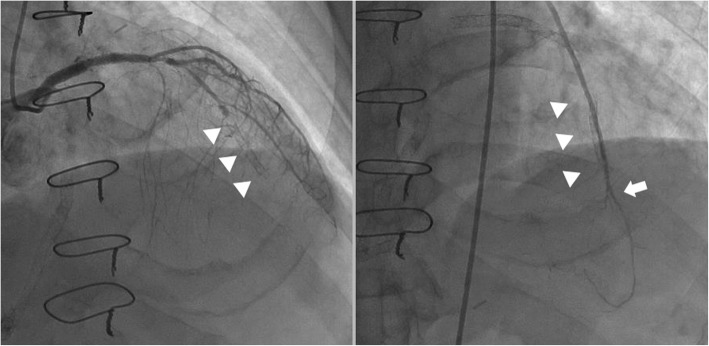
Illustrative case. A 71-year old man with three stents in the LAD underwent coronary artery bypass grafting including ITA to LAD. Two years later, the LAD had totally occluded at the stent that had been implanted distally (arrow heads, left image), limiting the revascularized area to the apical area (right image). A broad area of anterior ischemia had redeveloped. An arrow indicates the site of anastomosis of the ITA and LAD. ITA; internal thoracic artery, LAD; left anterior descending artery

PCI had a negative impact on graft flow only in ITA to LAD grafts, likely because PCI is indicated for LCX and RCA only when the vessel is sufficiently large. Possible mechanisms for reduced graft flow, higher rate of FI, and graft failure after PCI include the following. First, stenosis in the LAD may have been less severe in patients who had undergone PCI than in those who had not. Although, there was not a statistically significant difference in severity of stenosis and MLD, the incidence of angiographic competitive flow was higher in the PCI than in the no-PCI group. Second, revascularized areas were sometimes made smaller by sacrificing epicardial coronary vessels, such as the LAD, diagonal or septal branch (Fig. 2). A third possible mechanism is microvascular disease (MVD) distal to a PCI. It is widely accepted that PCI can cause microembolization of atherosclerotic debris or thrombus to distal myocardial tissue. Additionally, drug-eluting stents may adversely affect peripheral vascular function. Shin and colleagues reported that coronary segments distal to drug-eluting stents have more severe vasoconstriction than those distal to bare metal stents. These researchers suspected endothelial dysfunction in the myocardium distal to the treated vessel [13]. De Villa and colleagues have proposed normalized perfusion pressure, secondary inflammation, and platelet activation as possible mechanisms for MVD [14]. Fourth, patients who present with MVD are vulnerable to developing restenosis after PCI and therefore tend to be referred for CABG. De Villa and colleagues reported that lower coronary flow responses to adenosine and cold pressor tests are associated with restenosis after PCI [14]. Guidelines for revascularization recommend CABG only for proximal lesions of the LAD in patients with multivessel disease [15]. However, in our experience, graft flow is significantly reduced in ITA to LAD distal lesions with prior PCI and the incidence of FI and graft failure are as high as 32 and 12%, respectively. Both proximal and distal LAD stenosis should be carefully managed by the cardiovascular team, otherwise PCI to LAD distal lesions may cause MVD and compromise the efficacy of future CABG.

Recently, hybrid procedures comprising in situ ITA to LAD bypass grafting with drug-eluting stents for non-LAD vessels have been increasingly performed on patients at high risk of sternotomy [16, 17]. Rosenblum and colleagues reported finding no significant differences between such hybrid procedures and CABG using bilateral ITA over a mean duration of follow-up of 2.83 years and concluded that hybrid revascularization is effective in appropriately selected patients [18]. Patients are usually selected for hybrid revascularization when the morphology of LAD lesions contraindicates PCI [15, 19]. However, CABG is not necessarily appropriate in patients with morphology that contraindicates PCI, except for those with CTO. Even in vessels with CTO, small revascularized areas and a history of PCI have negative impacts on ITA to LAD grafts. Inversely, ITA grafting may be more beneficial than PCI in some patients with LAD in whom PCI would be appropriate. Moreover, the latest randomized study found no significant advantage of bilateral ITA over single ITA over a 5 year follow-up [20]. Detailed assessment of the suitability of coronary lesions for ITA or CABG and precise prediction of graft patency would contribute to improving outcomes of both hybrid revascularization and CABG using multiple arteries or both ITAs.

This study has the following limitations. First, it was retrospective and therefore not randomized. Second, we were unable to reliably examine FFR because it cannot be measured in target vessels with CTO and was not performed in other patients in some of the referral hospitals during the study period. Especially for in-stent stenosis, FFR or intravascular ultrasonography may be more reliable than angiography. However, these investigations had been performed only for selected vessels or patients, such as those with moderate stenosis or who were candidates for re-stenting. Difficulty in assessment or overestimation of in-stent stenosis may have introduced bias. Third, there may have been too few patients, especially for examining the effects of PCI in the LCX and RCA. We speculate that prior PCI in the LCX and RCA would show a statistically significant negative impact with greater numbers of patients and bypass grafts. Fourth, indications for PCI were not precisely defined because these procedures were performed using several different devices and techniques over more than a decade in a number of different hospitals. Of note, PCI has been performed more aggressively by cardiologists in Japan than in other countries. Fifth, myocardial flow demand in an area with a history of MI correlates with the extent of remaining viability. Unfortunately, viability had not been assessed by the appropriate specific preoperative investigations. Sixth, blood pressure or dose of catecholamine may have introduced biases in flow measurements. However, we could not precisely define these factors because this study was retrospective and the measurements had been taken intraoperatively. Finally, the most important limitation is the lack of a standard protocol for preoperative assessment and resultant uncertainty about all aspects of assessment and previous treatment.

## Conclusions

In conclusion, flow demand and myocardial status significantly influence graft flow and patency. The smaller the revascularized area, the more severe the stenosis must be to avoid FI. Moreover, too small a revascularized area or MVD, associated with PCI, can reduce the benefits of surgical coronary revascularization. When planning revascularization strategies, it is necessary to establish a logical and optimal way of taking into account factors associated with flow demand and microvasculature.

## Abbreviations

CABG: Coronary artery bypass grafting
FFR: Fractional flow reserve
FI: Flow insufficiency
GEA: Gastroepiploic artery
ITA: Internal thoracic artery
LAD: Left anterior descending artery
LCX: Left circumflex artery
MLD: Minimal luminal diameter
MVD: Microvascular disease
PCI: Percutaneous coronary intervention
RCA: Right coronary artery
SVG: Saphenous vein graft
TTFM: Transit-time flowmetry
